# Factors associated with community volunteering among adults over the age of 50 in Malaysia

**DOI:** 10.1371/journal.pone.0302220

**Published:** 2024-05-16

**Authors:** Rosnah Sutan, Khadijah Alavi, Siti Norain Sallahuddin, Mohd Rizal Abdul Manaf, Mohd Hasni Jaafar, Suzana Shahar, Khairul Nizam Abdul Maulud, Zaini Embong, Kamarul Baraini Keliwon, Ruzian Markom

**Affiliations:** 1 Department of Public Health Medicine, Faculty of Medicine, Universiti Kebangsaan Malaysia, Cheras, Kuala Lumpur, Malaysia; 2 Centre for Research in Psychology and Human Well-being, Faculty of Social Science and Humanities, Universiti Kebangsaan Malaysia, Bangi, Selangor, Malaysia; 3 Faculty of Health Sciences, Universiti Kebangsaan Malaysia, Cheras, Kuala Lumpur, Malaysia; 4 Department of Civil Engineering, Faculty of Engineering and Built Environment, Universiti Kebangsaan Malaysia, Bangi, Selangor, Malaysia; 5 Faculty of Economics and Management, Universiti Kebangsaan Malaysia, Bangi, Selangor, Malaysia; 6 Centre of International Law and Siyar (CILAS), Faculty of Law, Universiti Kebangsaan Malaysia, Bangi, Selangor, Malaysia; Alhussain University College, IRAQ

## Abstract

**Background:**

Community volunteering is defined as voluntary participation in activities and services to benefit the local community. It has potential benefits to promote social, physical, and mental well-being, and it enhances productive, healthy, and active aging. The tendency to volunteer varies across individuals and communities. There is limited knowledge of contributing factors influencing volunteering among Malaysian adults over the age of 50.

**Aims:**

The present study aims to assess the association of demographic, cultural, and social factors with volunteering among Malaysian adults over the age of 50.

**Methods:**

A cross-sectional study was conducted in 2020 involving 3,034 Malaysians aged 50 years and above across Malaysia, selected using a multi-stratified random sampling technique based on National Census 2020 data. A validated survey questionnaire to determine the demographic factor (age, sex, education level, employment status, health status, physical disability, and location of residence), cultural factor (ethnicity and religion), and social factor (social support, marital status, living arrangement, mode of transportation) that influence voluntary participation was distributed and collected. The association between these factors and volunteer participation was analysed using logistic regression models to identify significant predictors of voluntary participation among Malaysian adults over the age of 50.

**Results:**

A regression model indicates that living in rural areas (OR 2.03, 95% CI 1.63–2.53), having higher education level (Tertiary level: OR 2.77, 95% CI 1.86–4.13), being employed (OR 1.31, 95% CI 1.10–1.56), differences in ethnicity background (Chinese: OR 0.58, 95% CI 0.39–0.86) and ease of transportation (Driving private transport: OR 1.26, 95% CI 1.19–1.32; Public transport: OR 1.07, 95% CI 1.00–1.154) were significantly associated with volunteering with R^2^ Nagelkerke of 0.147.

**Conclusion:**

Recognising various factors towards community volunteering should be addressed by policymakers and volunteer organisations to increase volunteer participation from potential adults over the age of 50 in promoting healthy and active ageing.

## Introduction

The world’s population trend has been steadily growing toward an ageing population particularly in Asia and the Pacific region, with estimates suggesting that the number of people aged 60 years and older will double from 670 million in 2022 to 1.3 billion in 2050 [1]. As a developing country, Malaysia is no exception as it is fast transitioning into an ageing society with expected increase from 7% in 2021 to 15% by 2035 [2, 3]. Given the rapid demographic shift towards an ageing population, it presents significant challenges for public health as ageing is widely perceived to be connected with unproductivity, dependence, or loss in many aspects of life, portraying older people as vulnerable or incapable of contributing to society [4, 5]. Hence, the focuses of public health interventions for older adults has extended beyond physical health to include social engagement, with active ageing being a key international policy advocated by World Health Organisation (WHO) and the United Nations. As evidenced by successful volunteer programs in Japan and the United States, this policy promotes the involvement of older adults in social, economic, and cultural activities; emphasizing on the positive impact of volunteerism on promoting an active and productive ageing process [6–8].

The concept of volunteering refers to the United Nations General Assembly (UNGA) defined as a *“wide range of activities including mainstream forms of mutual support and self-help*, *formal service provision and other forms of civic engagement*, *done voluntarily*, *for the benefit of society as a whole and without economic retribution being the main motivating factor”* [5]. Getting involved with volunteer work allows adults over the age of 50 who will soon be at retirement age to plan to remain active, enhance their social connections, and embrace new roles, which significantly benefit their social, physical, and mental well-being [9]. Several studies have implied that regular volunteering improves the quality of life, cognitive function, and working memory, resulting in increased social interaction, improved health, and reduced depression [5, 10–13]. Additionally, a 5-year longitudinal study found that volunteering in later life lowers the risk of dementia compared to non-volunteers [14].

Promoting the overall well-being of older adults and healthy active aging aligns with Malaysia’s National Health Policy for Older Persons 2011 [15] and the proposed Malaysia Elderly Care Model (LTC) 2030 [16]. One of the strategies suggested to promote elderly health is focusing on community-based intervention programs led by trained older adult volunteers [16]. The concept of peer-led community-based intervention programs has also been shown to be more successful and influential [17–20]. Exploring the factors that predict the likelihood of older adults volunteering in community-based programs will assist stakeholders in targeting potential elderly groups to be trained as volunteers and indirectly act as peer support to others. To our knowledge, limited research is exploring the factors contributing to older adult community volunteering in Malaysia. Therefore, this study aims to determine the factors associated with volunteer participation among adults over the age of 50 in Malaysia.

## Method

### Study design

The data was obtained from a cross-sectional quantitative study of ‘Diagnostic Study to Develop an Integrated and Sustainable Elderly Care Model (LTC) in Malaysia by the year 2030’ [16]. The study was conducted throughout Malaysia involving 13 states and three Federal Territories according to the ethical approval granted for a period of 30 Jan 2020–29 Jan 2021.

### Study sample and sampling technique

Multi-stratified random sampling method was implemented. All Malaysians aged 50 years and above identified with the National Census Data 2020 were stratified by state and federal territories based on registered address. Malaysia has 14 states and two federal territories (counted as 16 states in the present study). Then, one district from each state was purposively selected as study site in which the selection based on the highest percentage of residing Malaysian aged 50 years and above. Selection of only one populous district for each state was to represent the primary needs of the elderly. In the Malaysian Census, the data was captured using the enumeration block. An enumeration block (EB) is a land area which is artificially created and consists of specific boundaries in which each EB has an average of 80 to 120 living quarters (LQ) with approximately 500 to 600 persons [2]. From the 16 selected districts, 48 urban EBs and 16 rural EBs were requested from the Department of Statistics Malaysia by stratified according to urban and rural areas with a ratio of three urban EB to one rural EB to represent the proportion or Malaysian urbanization status. Then, random sampling technique were used in selection of living quarters (LQ) from urban and rural EBs of each selected district. The division of total LQ from urban and rural EB was calculated based on the total population ratio of the selected districts in Malaysia according to the total sample size required. There were 2491 urban LQs and 573 rural LQs needed. If the selected living quarters could not be contacted or not consented to the study, living quarters on the right were selected first followed by left, front or back following the approach used in our Malaysian Census [2]. A member in the selected LQ who most closely meet the inclusion requirements were included in this study. At all points in time, written informed consents were taken and if the respondents were unable to provide information, then the information was obtained from the guardians. Malaysians aged 50 years and above who consented to participate were included for this study. The rationale of selecting participants age 50 years and above is to examine the pattern of volunteering in the pre-elderly group as well in elderly group to assess the needs and planning for successful ageing. Participants who were not at home at the time of data collection and who were non-Malaysians were excluded from the study. Only one respondent selected from each LQ. Further details of the study design and recruitment procedures were described in the Diagnostic Study to Develop an Integrated and Sustainable Elderly Care Model (LTC) in Malaysia by the year 2030 [16].

### Instrument

A self-administered questionnaire was distributed when conducting home visits to the selected living quarters. However, during the Movement Control Order was imposed following COVID-19 pandemic peak, google online forms were implemented instead of printed questionnaires and telephone calls were made to the selected living quarters. The questionnaire consists of five components: sociodemographic profile, living arrangement, voluntary participation, social support scale and preferences mode of transportation used. The content of the questionnaire has been validated by the experts and the reliability testing of the questionnaire was satisfactory (Kuder-Richardson Formula 20 coefficient was > 0.90 for dichotomous items).

### Measures

#### Dependent variable

For dependent variable, the evaluation would be any volunteering or voluntary participation within the past 12 months from time of data collection. In the study, participants were asked “Have you participate in voluntary activities within your community for the past 12 months?” and answer was recorded as dichotomous variable of ‘Yes’ or ‘No’.

#### Independent variables

This study examined the human, social, and cultural factors as well as age and sex, that are associated with voluntary participation as independent variables. The variables are; Age (*Age*: *50–59 years*, *Age*: *60–69 years*, *Age*: *70–79 years*, and *Age*: *80 years and above*), sex (*Male* and *Female*), location of residence based on permanent address (*Urban* and *Rural*), highest education level achieved (*Tertiary education*, *Secondary education*, *Primary education*, and *No formal education*), employment status (*Employed* and *Unemployed/Retiree/Homemaker*), current health status (*No multimorbidity*: no disease or has one chronic disease, and *Multimorbidity*: coexistence of two or more chronic diseases; Researcher suggested that when compared with people who only have one disease, people with multimorbidity are more likely to face difficulties in daily living activities [21], physical disability (*Yes* and *No*), ethnicity (*Malay*, *Chinese*, *Indian* and *Others*), religion (*Islam*, *Buddha*, *Hindu*, *Christian* and *Others*), marital status (*Married*, and *Never married*: including those who were divorced, widowed, separated or single), living arrangement (*Does not lives alone*: including those who lives with family members, spouse or at an institution, and *Lives alone*), social support score (nine items with four-point Likert Scale ranging from ‘Strongly disagree’ to ‘Strongly Agree’) and preferences on mode of transportation used (five-point Likert Scale ranging from ‘Always’, to ‘Never’ for each seven categories; *Private transport (Driver)*, *Private transport (Passenger)*, *Public transport*, *E-hailing/Taxi*, *Walking*, *Cycling*, and *Adapted transport*: includes ambulance or special-assisted vehicle).

### Data analysis

Data was analysed using Statistical package SPSS version 26. Normality test of the dataset distribution for scale variables was assessed based on skewness (-1 and +1) and kurtosis (-2 and +2). Descriptive analysis of the quantitative data was performed. Inferential statistics using bivariate analysis was performed between the independent variables and dependent variable (voluntary participation) to determine the association by calculating the chi-square (χ^2^) or comparing means with the independent Student’s *t*-statistic (t) depending on type of variables. Thirdly, multivariable logistic regression analysis was used to determine which factors predicted volunteering among adults over age 50 in Malaysia. Assessment of the multicollinearity of the independent variables were performed to ensure there were not correlated with each other. Linear relationship of independent variables to log odds were performed. In assessing the fitness of regression model, the Hosmer-Lemeshow test and Nagelkerke R-squared value were employed. Hosmer-Lemeshow test calculated chi-square statistics that indicates the discrepancy between observed and expected events within the model population. A low chi-squared value suggests that the model has a good fit [22]. Variables for the model were selected based on Backward LR method. Nagelkerke R-squared provide an estimation of the explained variance of the model.

### Ethics approval

This study was conducted according to the guidelines of the Declaration of Helsinki and approved by the Institutional Review Board (or Ethics Committee) of *Universiti Kebangsaan Malaysia* Research Ethics Committee (S190331-consultation project; UKM:PPI/111/8/JEP-2020-012) and approved on 30^th^ Jan 2020 for a study period of 30 Jan 2020–29 Jan 2021 with the title ‘A study of the profile and needs of people aged 50 years and above in integrated and sustainable long-term care for the elderly in Malaysia’.

## Results

### Descriptive analysis

In total, 3,034 older adults (aged 50 and above) responded to the questionnaire and the response rate was 99.0 percent. Descriptive characteristics are summarised in Table 1. Overall, there were more male respondents (53%) who participated in this study and majority of the respondents were from urban areas (84%). There were 39% respondents within age group of 50–59 years and 61% were aged 60 years and above. About 33% of respondents reported participating in voluntary activities within the past 12 months. Majority participated in religion-associated activities (88%) and community-related activities (82%) as shown in Table 2.

**Table 1:** Descriptive and bivariate analysis of volunteering and non-volunteering respondents (N = 3034).

Variable		n (%)	Voluntary participation n (%)		chi-squared statistic	*p*-value
			Yes (n = 1001)	No (n = 2033)		
Age group (in years)						
	50–59	1184 (39.0)	466 (39.4)	718 (60.6)	51.140	<0.001*
	60–69	1142 (37.6)	362 (31.7)	780 (68.3)		
	70–79	513 (16.9)	138 (26.9)	375 (73.1)		
	80 and above	195 (6.4)	35 (17.9)	160 (82.1)		
Sex						
	Male	1600 (52.7)	592 (37)	1008 (63)	24.589	<0.001*
	Female	1434 (47.3)	409 (28.5)	1025 (71.5)		
Location of residence						
	Urban	2532 (83.5)	780 (30.8)	1752 (69.2)	33.110	<0.001*
	Rural	502 (16.5)	221 (44.0)	281 (56.0)		
Marital status						
	Married	2210 (72.8)	791 (35.8)	1419 (64.2)	28.839	<0.001*
	Never married^a^	824 (27.2)	210 (25.5)	614 (74.5)		
Education level						
	Tertiary education	358 (11.8)	191 (53.4)	167 (46.6)	100.003	<0.001*
	Secondary education	1601 (52.8)	541 (33.8)	1060 (66.2)		
	Primary education	802 (26.4)	209 (26.1)	593 (73.9)		
	No formal education	273 (9.0)	60 (22.0)	213 (78.0)		
Employment status						
	Employed^b^	1481 (48.8)	597 (40.3)	884 (59.7)	70.085	<0.001*
	Unemployed/ Retiree/ Homemaker^c^	1553 (51.2)	404 (26.0)	1149 (74.0)		
Health status						
	No multimorbidity^d^	1640 (54.1)	591 (36.0)	1049 (64.0)	14.959	<0.001*
	Multimorbidity^e^	1394 (45.9)	410 (29.4)	984 (70.6)		
Physical disability						
	No	2947 (97.1)	986 (33.5)	1961 (66.5)	10.052	0.002*
	Yes	87 (2.9)	15 (17.2)	72 (82.8)		
Ethnicity						
	Malay	1755 (57.8)	640 (36.5)	1115 (63.5)	26.764	<0.001*
	Chinese	638 (21.0)	170 (26.6)	468 (73.4)		
	Indian	465 (15.3)	130 (28.0)	335 (72)		
	Others	176 (5.8)	61 (34.7)	115 (65.3)		
Religion						
	Islam	1875 (61.8)	671 (35.8)	1204 (64.2)	30.381	<0.001*
	Buddha	522 (17.2)	143 (27.4)	379 (72.6)		
	Hindu	403 (13.3)	101 (25.1)	302 (74.9)		
	Christian	220 (7.3)	84 (38.2)	136 (61.8)		
	Others	14 (0.5)	2 (14.3)	12 (85.7)		
Living arrangement						
	Does not live alone^f^	2758 (90.9)	917 (33.2)	1841 (66.8)	0.899	0.343
	Lives alone	276 (9.1)	84 (30.4)	192 (69.6)		
Social support^ (Range: 0–9)		7.2 (2.08)	7.4 (1.92)	7.1 (2.15)	-4.448^$^	<0.001**
Preference mode of transportation used^ (Range: 0–5)						
	Private transport^g^ (Driver)	3.2 (1.83)	3.8 (1.64)	2.9 (1.84)	-14.193^$^	<0.001**
	Private transport^g^ (Passenger)	3.2 (1.46)	3.0 (1.37)	3.3 (1.50)	4.868^$^	<0.001**
	Public transport	2.3 (1.39)	2.3 (1.32)	2.3 (1.43)	-1.791^$^	0.073
	E-hailing/Taxi	2.2 (1.35)	2.2 (1.27)	2.2 (1.39)	-0.187^$^	0.851
	Walking	2.2 (1.44)	2.2 (1.38)	2.2 (1.48)	0.043^$^	0.966
	Cycling	1.5 (1.09)	1.5 (1.02)	1.5 (1.13)	0.056^$^	0.956
	Adapted transport^h^	1.5 (1.04)	1.4 (0.96)	1.5 (1.08)	2.426^$^	0.015**

Note(s)

^a^Never married defined as single, divorced, widowed or separated.

^b^Employed defined as part-time and full-time workers.

^c^Homemaker includes housewife.

^d^No multimorbidity defined as individuals who have no disease or 1 chronic disease.

^e^Multimorbidity defined as individuals who have 2 or more chronic diseases.

^f^ Does not lives alone defined as either living with spouse, or family members, or in an institution, or other than stated.

^g^Private transport includes car or motorcycle.

^h^Adapted transport includes ambulance and special-assisted vehicles.

^Mean (SD). Social support is a mean of total possible range of 0–9. Preference mode of transportation used is a mean of total possible range of 0–5. SD = standard deviation

^$^Student’s t-statistic test. 95% CI = 95% confidence interval.

*significant p<0.05 (Pearson Chi Square).

**significant p<0.05 (student’s t-test, equal variances not assumed)

**Table 2:** Type of voluntary activities among those who volunteered (N = 1001).

Type of voluntary activities	Number of respondents n (%)
Religious activities	882 (88.1)
Community activities	816 (81.5)
Social visits	808 (80.7)
Political-related	542 (54.1)
Knowledge-related (teaching and learning)	517 (51.6)
Fundraising	483 (48.3)

### Inferential analysis

#### Factors associated with volunteering

A bivariate analysis using Chi-square test and independent Student’s t-test was performed for the independent variables to determine the relationship with volunteering in older adults as shown in Table 1. The result showed that voluntary participation was significantly associated with age, sex, location of residence, marital status, education level, employment status, health status, physical disability, ethnicity, social support, driving private transport, passenger of private transport and using adapted transportation.

#### Explanatory variables for volunteering in older adults

A multivariable logistic regression analysis was conducted to identify the explanatory factor for volunteering among older adults in the sample. A model was calculated based on the important variables selected by backward elimination (Likelihood Ratio) method. Multicollinearity was thoroughly assessed to ensure validity of regression model and each independent variable was below accepted Variance Inflation Factor threshold (VIF <10). The analysed model had a good fit according to the results of the Hosmer and Lemeshow test (chi-squared statistic = 13.773, *p =* 0.088) with R^2^ Nagelkerke of 0.147.

The results of the multivariable regression model is shown in Table 3. Location of residence, education level, employment status and mode of transportation were directly associated with volunteering. Specifically, older adults living in rural area were twice as likely to volunteer than those in urban areas (Urban: Reference; Rural: AOR = 2.03, 95% CI 1.64–2.53). Voluntary participation was also more likely among (1) those with tertiary education (No formal education: Reference; Tertiary education: AOR = 2.77, 95% CI 1.86–4.13), and (2) those who are employed (Unemployed/Retiree/Homemaker: Reference; Employed: AOR = 1.31, 95% CI 1.10–1.56). The findings also showed a significant association between older adult’s preferences mode of transportations used and volunteering. The positive predictor towards volunteering were the older adults who drives their own vehicle, either a car or motorcycle (AOR = 1.26, 95% CI 1.19–1.32) and those who used public transport (AOR = 1.07, 95% CI 1.00–1.14). Whereas older adults who rely on another person’s car or motorcycle (AOR = 0.93, 95% CI 0.88–0.99), and those who preferred to used adapted transportation such as ambulance or special-assisted vehicle (AOR = 0.88, 95% CI 0.81–0.96) were less likely to participate in voluntary activities.

**Table 3:** Factors associated with volunteers and non-volunteers in older adults.

Variable		Crude OR (95% CI)	*p*-value	Adjusted OR (95% CI)	chi-squared statistic* (df)	*p*-value
Age group						
	50–59	2.97 (2.02, 4.36)	<0.001	1.36 (0.90, 2.06)	2.08 (1)	0.150
	60–69	2.12 (1.44, 3.12)	<0.001	1.24 (0.82, 1.87)	1.04 (1)	0.308
	70–79	1.68 (1.11, 2.55)	0.014	1.35 (0.87, 2.07)	1.81 (1)	0.179
	80 and above	1.00		1.00		
Sex						
	Female	1.00				
	Male	1.47 (1.26, 1.72)	<0.001			
Location of residence						
	Urban	1.00		1.00		
	Rural	1.77 (1.45,2.15)	<0.001	2.03 (1.63,2.53)	40.32 (1)	<0.001
Marital status						
	Never married^a^	1.00		1.00		
	Married	1.63 (1.36, 1.95)	<0.001	1.21 (0.99, 1.46)	3.54 (1)	0.060
Education level						
	No formal education	1.00		1.00		
	Primary education	1.25 (0.90, 1.74)	0.179	1.19 (0.84, 1.69)	0.94 (1)	0.332
	Secondary education	1.81 (1.34, 2.46)	<0.001	1.38 (0.98, 1.95)	3.38 (1)	0.066
	Tertiary education	4.06 (2.85, 5.78)	<0.001	2.77 (1.86, 4.13)	24.00 (1)	<0.001
Employment status						
	Unemployed/ Retiree/ Homemaker^b^	1.00		1.00		
	Employed^c^	1.92 (1.65, 2.24)	<0.001	1.31 (1.10 1.56)	9.41 (1)	0.002
Health status						
	Multimorbidity^d^	1.00		1.00		
	No multimorbidity^e^	1.35 (1.16,1.58)	<0.001	1.13 (0.96, 1.34)	2.17 (1)	0.141
Physical disability						
	Yes	1.00		1.00		
	No	2.41 (1.38, 4.23)	0.002	1.66 (0.92, 3.01)	2.81 (1)	0.094
Ethnicity						
	Malay	1.08 (0.78, 1.50)	0.634			
	Chinese	0.69 (0.48, 0.98)	0.037			
	Indian	0.73 (0.51, 1.06)	0.098			
	Others	1.00				
Religion						
	Islam	3.34 (0.75, 14.99)	0.115	0.71 (0.47, 1.08)	2.61 (1)	0.106
	Buddha	2.26 (0.50, 10.24)	0.289	0.58 (0.39, 0.86)	7.19 (1)	0.007
	Hindu	2.01 (0.44, 9.12)	0.367	0.84 (0.58, 1.21)	0.88 (1)	0.350
	Christian	3.71 (0.81, 16.97)	0.092	1.00		
	Others	1.00				
Living arrangement						
	Lives alone	1.00				
	Does not lives alone^f^	1.14 (0.87, 1.49)	0.343			
Social support (Range: 0–9)		1.09 (1.05, 1.13)	<0.001			
Mode of transportation (Range: 0–5)						
	Private transport^g^ (Driver)	1.34 (1.29, 1.41)	<0.001	1.27 (1.19, 1.32)	74.58 (1)	<0.001
	Private transport^g^ (passenger)	0.88 (0.84, 0.93)	<0.001	0.93 (0.88, 0.99)	4.90 (1)	0.027
	Public transport	1.05 (0.99, 1.11)	0.081	1.08 (1.00, 1.14)	4.27 (1)	0.039
	E-hailing/Taxi	1.01 (0.95, 1.06)	0.856			
	Walking	1.00 (0.95, 1.05)	0.966			
	Cycling	1.00 (0.93, 1.07)	0.956			
	Adapted transport^h^	0.91 (0.85, 0.99)	0.02	0.88 (0.81, 0.96)	8.59 (1)	0.003

Note(s)

^a^Never married defined as single, divorced, widowed or separated. ^b^Employed defined as part-time and full-time workers. ^c^Homemaker includes housewife. ^d^No multimorbidity defined as individuals who have no disease or 1 chronic disease. ^e^Multimorbidity defined as individuals who have 2 or more chronic diseases. ^f^ Does not lives alone defined as either living with spouse, or family members, or in an institution, or other than stated. ^g^Private transport includes car or motorcycle. ^h^Adapted transport includes ambulance and special-assisted vehicles.

*Wald statistics. 95% CI = 95% confidence interval.

Crude OR = Crude Odds Ratio, Adjusted OR = Adjusted Odds Ratio.

Dependent variable: Volunteering as reported by respondents. Significant at 0.05 level

Between different ethnic groups with ‘Others’ as reference, significant findings were only found in Chinese ethnic group. Older Chinese adults were 40% less likely to volunteer compared to their counterparts (Others: Ref; Chinese: AOR = 0.58, 95% CI 0.39–0.86). Age, health status, physical disability and marital status were not shown to be significantly associated with volunteering among older adults in this study.

## Discussion

The aim of this study was to determine the factors associated with community volunteering among adults age above 50 in Malaysia. Volunteering as participants in any local group or organisation, or as a trained volunteer in a community intervention programme, can promote overall well-being and encourage productive active ageing [23]. Based on the Human Capital Theory [24, 25], the predictors that influence voluntary participation consist of human, social and cultural capital as well as exogenous factors.

Multivariable models were developed and tested in the present study to determine the relative importance of various factors associated with voluntary participation among older adults in Malaysia. Of all the factors studied, urban-rural location of residence, education level and employment status were found to be significantly important. Older adults living in rural areas were twice as likely to volunteer than in urban areas. Those who received prior school education were more likely to volunteer and the tendency increases the higher the education level they experienced. Older adults working full-time or part-time volunteer more than unemployed or retired. In addition, older adults who can drive cars or ride motorcycle were positively associated with engagement in volunteering, as well as those who used public transport.

Education and employment have consistently been found to be predictors of volunteering. Level of education does not only measure one’s knowledge, but it also related to employment status. The likelihood to volunteer among those with higher level of education and working were consistent with previous research that indicate volunteerism is facilitated by greater human capital. The higher the level of education one’s received, the more likely they would participate as volunteer [26–30]. Education promotes volunteering by increasing awareness of concerns, building self-confidence, and having adequate literacy skills for information seeking [24, 29]. Regarding employment, there was a greater likelihood of volunteering among those working compared to those no longer working [26]. The connection between work and volunteering is a form of social integration and enables people to develop more civic skills, both of which influence the potential for volunteering [24].

Another significant finding was the increased likelihood of volunteering in older adults living in rural areas as opposed to urban ones. The finding varies across previous studies and were inconsistent; one previous study showed rural elderly were more likely to engaged in religious and community activities than those from urban area [31] and few studies showed that there was no significant association between urban-rural differences in volunteering [32, 33]. It was previously assumed that those living in urban areas have more opportunities to volunteer as they are more expose to formal organisation [11, 26]. However, a study conducted in Belgium, showed that neighbourhood connectedness and neighbourhood satisfaction played a significant role as predictors in volunteering rather than exposure to formal organisation [33]. People living in rural have more neighbourhood connectedness as the rural residents interact more with their neighbours, which leads to more likelihood of participating in community activities than urban residents [31]. Given the significant association between volunteering and rural respondents in the present study, targeting rural communities to initiate and promoting peer-led community intervention program may be more effective and successful.

In terms of significant findings on likelihood in volunteering in older adults who drives, as well as uses public transportation, this is consistent with previous research that found older adults who primarily use spontaneous and accessible mode of transportation, including those who drive or use public transportation, have higher level of overall community participation [34]. Participation in voluntary activities may be hampered by lack of transportation options, especially for those without access to personal vehicles. Therefore, addressing transportation barriers may enhance voluntary participation in older adults.

This study also showed the different likelihood of volunteering across different racial groups. Malays are the majority ethnic group in Malaysia (69.9%), followed by Chinese (22.8%) and Indian (6.6%) [2]. Our research found that older Chinese adults were 40% less likely to volunteer than their counterparts. This finding was consistent with a similar study by Teh et al. (2023) on active engagement among older Malaysian that found Chinese elderly were less likely to be involved in community activities and religious activities when compared with Malays [31]. Cultural and community norms may have influenced the volunteering differences between different ethnic groups as different ethnic communities have varying cultural values and motivations towards community involvement and different socioeconomic backgrounds [35]. Similar results on ethnicity significance on voluntary participation are supported by previous study in the United States that have found Asians, Hispanic and African Americans volunteered less than White Americans [26, 36]. As Malaysia is a multi-racial country, acknowledging and respecting the differences in volunteering across different racial groups may assist organisations in focusing their activity toward specific groups to encourage participation.

In this study, age is not a barrier to community volunteering as no significant association was found when adjusted to confounders. Although there have been consistent findings of less likelihood to volunteer as age increase [27, 37, 38], age should not be seen as a hindrance to encourage participation in community programmes. This study also demonstrates no association between health status and physical disability with volunteering when adjusted in final regression model although in the initial analysis both variables showed significant result. The change in significance suggests that other variables included in the model may have confounded initial associations, which, when corrected for, decreased the direct impact of health status and physical disability on volunteering. Despite the growing evidence of good physical health being positively associated with volunteering among older adults [9, 11, 26, 30], it was not shown to be significant in this study.

Sex and social support also showed significant direct relationship with volunteering when unadjusted to confounders. Sex differences influence the types of volunteering activities, motivation for volunteering and frequency of participation. Studies found that volunteering is less likely in male elderly compared to female [26, 27]. There may be different reasons for women and men to participate in volunteering, and they may also encounter different barriers, such as caregiving responsibilities or social expectations. Similarly, social support can enhance or hinder volunteer activities as it can provide necessary resources, encouragement or network connections that enable volunteering. On the other hand, a lack of social support may make participation more difficult.

One of the limitations is that the study only considers the patient’s objective health. The subjective health, such as psychological well-being and quality of life was not represented. Evidence showed that higher health optimism, which occurs when an individual’s subjective health is better than objective health, correlates with higher propensity to volunteer [27]. A recommendation is to include self-rated health as an alternative proxy for actual health and poor health. There are few other limitations to this present study. First, data were based on self-rated methods. Therefore, there is a potential to response bias. For example, the respondent may choose extreme rating categories to orient themselves to societal standards in order to display socially desirable responses. Secondly, the methods did not measure the frequency of participation in voluntary activities or voluntary hours, as 201 or more annual hours of volunteering, which equals nearly 4 hours per week, is a significant commitment of personal time and effort [9].

Future recommendation for research on association between neighbourhood connectedness and satisfaction with voluntary participation are worth exploring as there is growing evidence that connectedness to one’s neighbourhood increase the likelihood of becoming potential volunteer [33].

## Conclusion

This study contributed to building understanding about older adults who have higher potential to volunteer. It is evident from the analysis that older adults who lives in rural areas, with higher education, who are working and have accessible transportation were more involved in voluntary activities. With the information obtained from this study, program leaders and volunteer organisation could take appropriate measures in identifying older adults who could lead community-based health programme voluntarily. Policy makers could make necessary adjustments to current programs and plan specific strategies to increase participation from potential older adults in promoting healthy and active ageing.
